# Development of a Core Outcome Set of Domains to Evaluate Acute Pain Treatment After Lumbar Spine Surgery: A Modified Delphi Study

**DOI:** 10.1002/ejp.4784

**Published:** 2025-01-13

**Authors:** Ilse H. van de Wijgert, Kris C. P. Vissers, Maaike G. E. Fenten, Akkie Rood, Regina L. M. van Boekel, Miranda L. van Hooff

**Affiliations:** ^1^ Department of Anesthesiology Sint Maartenskliniek Nijmegen The Netherlands; ^2^ Department of Anesthesiology, Pain and Palliative Medicine Radboud University Medical Center Nijmegen The Netherlands; ^3^ Department of Research Sint Maartenskliniek Nijmegen The Netherlands; ^4^ Department of Orthopaedic Surgery Sint Maartenskliniek Nijmegen The Netherlands; ^5^ Research Department of Emergency and Critical Care HAN University of Applied Sciences Nijmegen The Netherlands; ^6^ Department of Orthopaedic Surgery Radboud University Medical Center Nijmegen The Netherlands

## Abstract

**Background:**

After lumbar spine surgery, a Core Outcome Set (COS) for acute pain is essential to ensure that the most meaningful outcomes are monitored consistently in the perioperative period. The aim of the present study was to consent on a COS for assessing the efficacy of acute pain management for patients undergoing lumbar spinal surgery.

**Method:**

A modified Delphi procedure was conducted among a national (Dutch) expert panel. External endorsement of the final COS was conducted among an international panel of anaesthesiologists and the Dutch chronic pain patient association.

**Results:**

A panel of 35 experts representing 10 stakeholder groups, including orthopaedic surgeons, anaesthesiologists, patient representatives, physician assistants, researchers, a neurosurgeon, nurses, and a psychologist, took part in the Delphi procedure. Five outcome domains reached consensus for inclusion in this COS. This COS contains the following domains: pain intensity, analgesic use, early mobilisation, length of stay, and adverse events. Of an international panel of 27 key opinion leaders, 77% agreed on the final COS. The patient association also consented to the final COS.

**Conclusions:**

A COS to evaluate acute pain treatment after lumbar surgery is proposed after national Delphi consensus rounds and (international) external endorsement. Future research should focus on determining suitable measurement instruments, assessing feasibility, validation, and implementation of the COS in daily clinical practice and research.

**Significance:**

This research proposes a clinically relevant spine‐specific core outcome set (COS) of domains focusing on the acute postoperative phase (until 30 days). This is the first COS for evaluation of acute pain after lumbar spine surgery.

**Abbreviations**
COMET: core outcome measures in effectiveness trials
COS: core outcome set
COSMIN: consensus‐based standards for the selection of health measurement instruments
LSS: lumbar spine surgery
METC: medical ethical research committee
NRS: numeric rating scale
PPSP: persistent postsurgical pain
WMO: human subjects act

## 1 Introduction

Lumbar spine surgery (LSS) is associated with severe acute postoperative pain in more than 50% of the patients (Nielsen et al. 2014). The development of acute postoperative pain after LSS is associated with multiple risk factors, such as preexisting chronic pain, related analgesic use, and emotional distress (Gilron, Kehlet, and Pogatzki‐Zahn 2019). Hence, inadequate relief of acute pain is a consistent predictor of the development of persistent postsurgical pain (PPSP) (Kehlet, Jensen, and Woolf 2006; Rosenberger and Pogatzki‐Zahn 2022). PPSP is defined as ‘pain that persists after the surgical wound has healed and lasts for more than 3–6 months after surgery’ (Kehlet, Jensen, and Woolf 2006). PPSP in turn, contributes to long‐term opioid use (Chou et al. 2018), with one in three patients using opioids at least 6 months after LSS (Vraa et al. 2022).

Different unidimensional pain scores have been shown to be valid for the measurement of acute postoperative pain intensity, such as the Numeric Rating Scale (NRS) (Gagliese et al. 2005). However, these pain scores alone are not reflecting the multidimensional aspects of acute pain nor the difference between acute and chronic pain (Hjermstad et al. 2011). Additional outcome domains should be considered to determine acute pain after LSS (Van Boekel et al. 2017). A recent scoping review identified a wide variety in outcome measurement across intervention studies on perioperative pain in LSS, which reflects the lack of global consensus on this topic (van de Wijgert et al. 2022). Harmonisation of outcome domains and measurement instruments across research and clinical practice is required to assess if a treatment is effective or not.

A core outcome set (COS) is a consensus‐based, minimal and standardised set of outcomes considered useful in clinical practice to enhance the quality and safety for patients (Kirkham et al. 2017). As such, they can encourage that the most meaningful outcomes are routinely evaluated in all patients undergoing LSS (Clarke et al. n.d.). A COS was developed by the IMI‐PROMPT research group to assess effectiveness in acute pain management after four different surgical procedures (Pogatzki‐Zahn et al. 2021). This generic COS provides input concerning important outcomes after other types of surgery and is a valuable foundation for further spine‐specific COS development. A spine‐surgery‐specific COS is deemed necessary to improve the total quality of care of surgery and perioperative anaesthesiology to result in the best possible quality of life perioperatively and, ultimately, in the longer term. With this COS we aim to facilitate adequate and standardised evaluation of anaesthesia and pain management in the early acute postoperative phase until 30 days after LSS by defining what to measure (Van Boekel et al. 2019; Guay and Kopp 2016).

The aim of this study was to develop the first dedicated COS for acute pain management for patients undergoing LSS. This COS includes outcome domains that matter to patients and the involved perioperative health care professionals and is based upon existing literature and expert panel consensus.

### 1.1 Scope

The new COS will be of value for patients with (chronic) back and/or leg pain, with visible (degenerative) lumbar spine pathology (i.e., herniated nucleus pulposus, stenosis, or spondylolisthesis), and unsuccessful conservative treatment options leading to elective LSS. This COS is applicable during the perioperative hospital phase until 30 days after LSS and is an addition to a COS that includes outcomes for longer‐term evaluation (from 6 to 24 months) (Chiarotto et al. 2015; Clement et al. 2015).

## 2 Methods

### 2.1 Protocol/Registry Entry

This study followed partly the recommendations for the development process of a COS provided by the Core Outcome Measures in Effectiveness Trials (COMET) initiative (Williamson et al. 2012). The study protocol was registered in the COMET database (number 2053) (COMET Initiative 2022).

### 2.2 Participants Delphi Panel

Dutch multidisciplinary stakeholders, with a minimum of 3 years' experience in care for and/or research into patients undergoing LSS, were approached via mail to participate as a panellist. Panellists were recruited by approaching orthopaedic surgeons, neurosurgeons, and anaesthesiologists from spine‐related hospitals in the Netherlands. Snowball sampling was established by asking the surgeons and anaesthesiologists to invite physiotherapists, recovery nurses, acute pain nurses, and other possibly interested colleagues. Participants consented to be a panellist and to abide by the Delphi procedure by confirmation via this mail.

Patient representatives who underwent LSS were included via the official patient participation panel (STAP panel, Sleutel Tot Actief Participatiebeleid, https://www.maartenskliniek.nl/research‐innovation/stap) from the Sint Maartenskliniek (Nijmegen, the Netherlands) to improve the clinical relevance of the COS. The patient representatives were informed by the coordinating researcher before the start of the Delphi panel to assure sufficient understanding of the Delphi questionnaires. All patient representatives had previously worked in health care, which made it possible to bring all participants to the same level and present the same Delphi questionnaires to the entire panel.

All panellists were required to complete a round in order to participate in the consecutive rounds. If not completed, a panellist was marked as a drop‐out, with the exception for round 3 due to the logistic challenges of a live round.

For the external endorsement round, a group of international anaesthesiologists was invited through email. Anaesthesiologists are considered the most experienced in the evaluation of acute pain management and the main responsible specialists for the management of acute postoperative pain. The project team selected this expert panel based on prior published articles on this subject and expertise.

Furthermore, as the patient's perspective is considered of pivotal importance, the Dutch chronic pain patient association was reached out to for their additions and/or consent to the final COS. Their perspective, considerations, and recommendations were considered.

### 2.3 Information Sources

An extensive preparatory phase took place before the start of this Delphi process. An initial list of outcomes was collected through a scoping review of outcomes used for evaluation of acute pain treatment after elective LSS, which was published separately (van de Wijgert et al. 2022). This initial list consists of 11 outcome domains. Information on how outcomes were selected, dropped, and combined can be found in this manuscript. All outcome domains were presented in Dutch to the Delphi panellists in the explanatory material, along with definitions, examples, and references. Explanatory material and hyperlinks were made available via a website or spreadsheet. Anonymous feedback reports were created and served as input for the subsequent Delphi round.

### 2.4 Outcome Scoring

Relevance was rated on a 9‐point Likert scale, ranging from 1 (strongly irrelevant) to 9 (strongly relevant) (Lange et al. 2020; De Meyer et al. 2019). ‘Relevance’ in the context of the development of this study was described as follows: ‘How much information does an outcome domain give about the postoperative recovery after lumbar spine surgery? How much does it say about the efficacy of the pain treatment?’ In the second round, panellists were asked to confirm whether they agreed with the consensus or rejection for the domains with consensus or rejection in the first round by voting agree/disagree/don't know. In the third round, outstanding issues were discussed, after which panellists were asked to vote for inclusion or exclusion. Voting was performed by means of raising (digital) hands for inclusion of the outcome domain. In the fourth round, the final (preliminary) COS was presented, and panellists were asked to vote for acceptance of the COS by voting yes or no.

### 2.5 Consensus Definition

Consensus on a topic for Rounds 1 and 2 was reached with ≥ 75% agreement among participants, scoring the topic with a 7–9 on the Likert scale. Topics were rejected when ≤ 30% of participants rated the topic as relevant (scores 7–9) or when ≥ 75% of the panellists agreed on the irrelevance of a specific topic (scores 1–3) (Diamond et al. 2014). Topics with no consensus, that is, between 30% and 75%, were presented in the subsequent round with additional information. The cut‐off for final inclusion in the COS was ≥ 75% agreement; the cut‐off for final rejection is ≤ 30% agreement for inclusion or ≥ 75% agreement for rejection. If a topic did not reach consensus after three rounds, it was rejected from the COS. A cut‐off of ≥ 75% agreement was used for the final agreement of the COS (Round 4) and for external endorsement.

### 2.6 Consensus Process

*Modified Delphi procedure*–consensus was pursued in four rounds (in Dutch) Meijer et al. (2003):

- Round 1—online: the panellists were asked to rate the initial outcome domains on ‘relevance’. To rate these topics, a 9‐point Likert scale was used. Participants could also add domains with supporting evidence.
- Round 2—online: the topics that did not reach consensus in Round 1 were presented again along with additional information gathered by the project team. The panellists were asked to rate the domains using the same 9‐point Likert scales as in the previous round. For the domains with consensus or rejection in the first round, panellists were asked to confirm whether they agreed with the consensus or rejection.
- Round 3—hybrid (live and digital) meeting: a sample of the panel participated in a video conference meeting on 20 December 2022. The goal was to establish consensus on the COS. The meeting had a formal character and a predefined structured schedule in which items with consensus in Round 2 were confirmed or rejected. Consensus was reached by voting by means of raising (digital) hands. Outstanding issues were discussed, after which panellists were asked to vote for inclusion or exclusion of the domain in the COS. Finally, the final (preliminary) COS was presented, and panellists were asked to vote for acceptance of the COS (yes/no).
- Round 4—online confirmation round: all panellists were invited to give their (dis)approval (yes/no) on the COS established in Round 3 to ensure broad support.

Questionnaires for each round were presented in the LimeSurvey GmbH online survey tool. Panellists had 2 weeks in between the rounds to complete the survey. Panellists received a reminder after 1 week and in the last days before the deadline. The final COS was translated to English by an official medical translator.

*External endorsement*—this online round was conducted to create support for future uptake of the COS among an international group of anesthesiologists. The external panellists were presented the final COS and were asked for agreement (yes/no) with the overall COS.

The Dutch chronic pain patient association (Pijn Patiënten naar 1 Stem, https://www.pijnpatientennaar1stem.nl/) was also presented the final COS and were asked whether or not they consent with the COS and if they have any additions.

### 2.7 Ethics and Consent

A waiver was obtained by the medical research ethics committee, METC Oost‐Nederland, as this study is not subject to the Medical Research Involving Human Subjects Act (WMO) (reference 2022–13648).

### 2.8 Project Team

This study was performed by an independent project team, not participating in the Delphi rounds, consisting of a methodologist (M.L.H.), a pain specialist (K.C.P.V.), a spine surgeon (A.R.), an anaesthesiologist (M.G.E.F.), and a physician‐junior researcher (I.H.W.). A.R. moderated the third hybrid meeting.

## 3 Results

### 3.1 Participants

In this study, a total of 35 Dutch participants participated in and completed round 1, of which 14 were female (40%). The panel consisted of 10 different stakeholder groups and represented 14 hospitals. Almost half (49%; 16/35) had more than 10 years' experience in the field of LSS (Table 1). A total of 32 panellists completed Round 2 (91%; 32/35), 11 panellists Round 3 (31%; 11/35), and 28 panellists Round 4 (80%; 28/35). Details on relevant panel characteristics per Delphi round are presented in Data S1.

**TABLE 1:** Overview of stakeholder groups that participated in the Delphi panel (rounds 1–4).

Stakeholder group (*n* = 35)	*N* (%)
Orthopaedic surgeon (spine surgeon)	13 (37%)
Anaesthesiologist	10 (29%)
Patient	3 (9%)
Physician assistant	2 (6%)
Researcher	2 (6%)
Neurosurgeon	1 (3%)
Recovery nurse	1 (3%)
Psychologist	1 (3%)
Acute pain service nurse	1 (3%)
Nurse practitioner	1 (3%)
Years of experience	
> 10 years	16 (49%)
5–10 years	14 (42%)
3–5 years	3 (9%)
Gender	
Male/Female	21/14 (60%/40%)

For the external endorsement, an international panel was formed of 27 worldwide key opinion leaders who replied to the questionnaire, consisting of 25 anaesthesiologists, a neurologist, and a health outcomes researcher. Of the external panel, 14% (5/27) were specialised in both acute and chronic pain medicine, 37% (10/27) in acute pain medicine, and 41% (12/27) were specialised in chronic pain medicine. The majority of this panel represented Europe (56%; 15/27). Other participants represented Asia (15%; 4/27), North America (11%; 3/27), Australia (11%; 3/27), and Africa (7%; 2/27).

### 3.2 Outcomes

**FIGURE 1:**
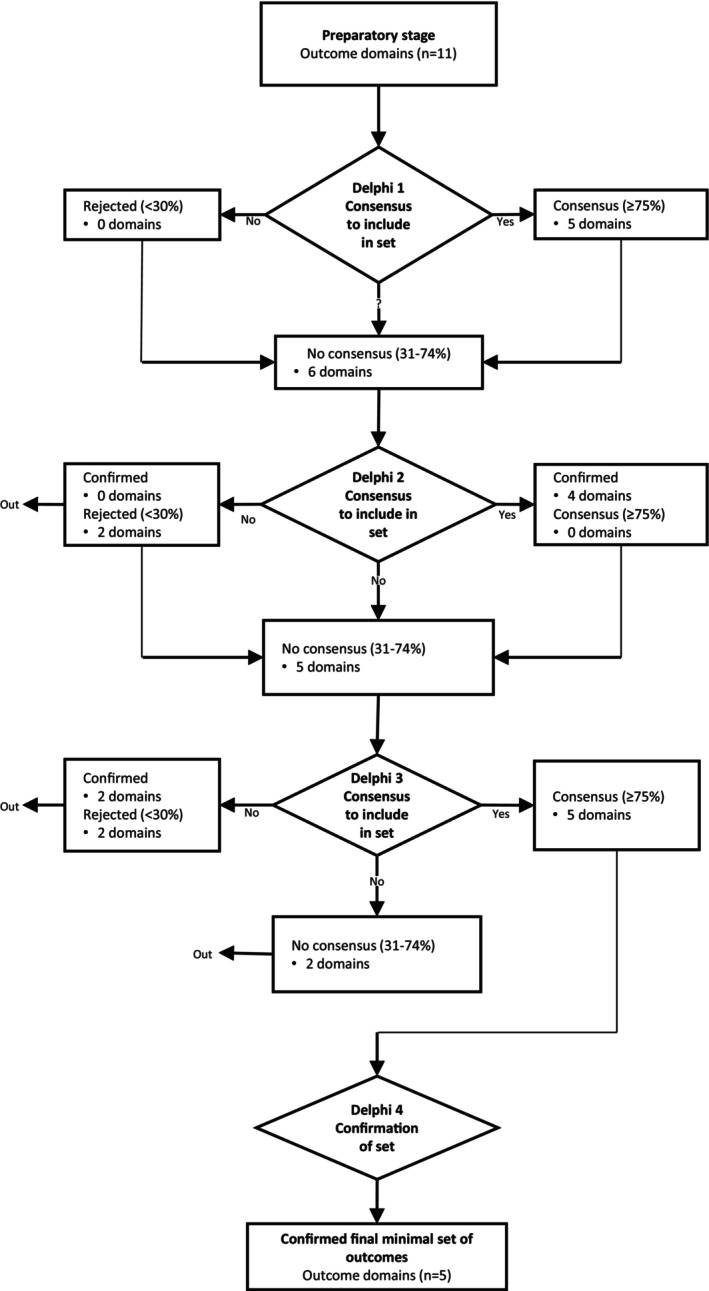
Flow of results throughout the modified Delphi procedure for outcome domains. The threshold for consensus was set at > 75% agreement. Items with < 30% agreement for two subsequent rounds were excluded from the COS. Items with 31%–74% consensus were made available again for voting in the subsequent round.

Figure 1 shows the flow chart of the selection process for outcome domains agreed upon for the COS. The initial list of outcomes from the preparatory phase is presented in Table 2, along with the consensus process for the individual items. The more detailed results of the voting procedure are presented per round in Data S2.

**TABLE 2:** Initial list of outcome domains and the consensus process.

Outcome domains	Round 1	Round 2	Round 3	Round 4
1. Adverse events	C	C	C	C
2. Pain intensity	C	C	C	C
3. Analgesic use	C	C	C	C
4. Early mobilisation	C	C	C	C
5. Quality of life	C	I	X	X
6. Length of stay	I	I	C	C
7. Daily functioning	I	I	I	X
8. Quality of recovery	I	I	I	X
9. Patient satisfaction	I	I	I	X
10. Physical function	I	X	X	X
11. Outcome domain: Quality of Sleep	I	X	X	X

Abbreviations: C, consensus reached; I, no consensus reached (inconclusive); X, threshold for exclusion.

#### 3.2.1 Delphi Round 1

In the first round, the initial list of outcomes identified in our scoping review (*n* = 11, included in Table 2) was rated on their ‘relevance’ (van de Wijgert et al. 2022). Of the 11 initial outcome domains, five reached consensus after the first round: adverse events, pain intensity, analgesic use, early mobilisation, and quality of life. No outcome domains were yet rejected from the COS (Figure 1). No outcome domains were added by the participants to the initial list of outcomes.

#### 3.2.2 Delphi Round 2

In the second round, four out of five domains agreed upon after round 1 were confirmed for inclusion: adverse events, pain intensity, analgesic use, and early mobilisation; the domain quality of life did not reach consensus for inclusion in the COS. The question arose whether quality of life is relevant or feasible for the acute postoperative phase. A panellist stated, ‘It is extremely important to measure quality of life, but it might be too early in this phase.’ The panellists agreed to list this domain for discussion in Round 3. Two other domains, physical function and quality of sleep, were rejected because, according to the panel, these do not measure the effectiveness of pain treatment.

#### 3.2.3 Delphi Round 3

In the third round, the preliminary COS was established. The panel consented on the following outcome domains: pain intensity, analgesic use, early mobilisation, length of stay, and adverse events. Domains that were rejected were quality of life (10% consensus), physical function (17%), and quality of sleep (23%). Outcome domains that reached ‘no consensus’ and were therefore not included in the COS were daily functioning (30% consensus), patient satisfaction (60%), and quality of recovery (50%).

A few domains were extensively discussed in this third round. Firstly, the domain patient satisfaction was deemed ambiguous and invalid to reflect patient satisfaction. One panellist stated, ‘It is a difficult domain because patient satisfaction can be influenced by a lot of factors (i.e., snoring roommate) that do not relate to the pain treatment of this specific patient.’ The panel agreed that a suitable and specific measurement instrument has to be validated before this domain can be included in the COS. Furthermore, the domain quality of life was rejected (10% consensus) because panellists agreed it is too early to measure this domain in the acute postoperative phase extensively. The domain quality of recovery was thought to be a possible early indicator of quality of life. This idea is not convincingly supported by scientific literature, as resulted from the scoping review that was performed prior to this Delphi procedure. The domain was only used in 5/75 articles (7%), and the specific QoR‐15 questionnaire only once. Therefore, this domain did not reach consensus for inclusion. Lastly, the domain of daily functioning did not reach consensus because the panel agreed that it is influenced by multiple factors in addition to pain.

#### 3.2.4 Delphi Round 4

In the fourth round, the confirmation round, 89.3% (25/28) of the panel agreed with the COS (Table 3).

**TABLE 3:** Core outcome set to evaluate acute pain treatment after lumbar spine surgery.

Domains	Definition	% Agreement for inclusion (round 4)
Pain intensity	Pain score at rest, in action, and interference of pain with daily activities (e.g., sleep)	94% Agree
		0% Disagree
		6% Don't know
Analgesic use	Analgesic use for back and/or leg pain, or pain in the operation area	97% Agree
		0% Disagree
		3% Don't know
Early mobilisation	The degree of mobilisation a patient has to achieve before hospital discharge, as well as the time it takes before a patient is independent in activities of daily living (ADL)	88% Agree
		3% Disagree
		9% Don't know
Length of stay	Length of hospital stay after spinal surgery	80% Agree
		20% Disagree
		0% Don't know
Adverse events	Any (serious) adverse event occurring in the first 30 days after surgery	97% Agree
		0% Disagree
		3% Don't know

#### 3.2.5 External Endorsement

##### 3.2.5.1 Anaesthesiologists

For the external endorsement round, 77.8% (21/27) agreed on the COS as established in the modified Delphi procedure, reaching the threshold for acceptance of the COS; 22.2% (6/27) disagreed. Arguments for disagreement were not including quality of life and functioning scores (*n* = 3). Furthermore, it was stated that the NRS has fallen out of favour in many places (*n* = 2).

##### 3.2.5.2 Patients

The Dutch chronic pain patient association consented with the final COS. Their view was that it is a good COS, and they had no additions to it. They did provide recommendations on the way of communication to the patient and good observations on these matters. For ‘length of stay’, it is important to consider possible difficulties in transfers to a nursing home or preoperative home situation. For complications, it is important to educate the patients on recognising those and when to alarm.

## 4 Discussion

The goal of this study was to create the first core outcome set (COS) on which outcome domains should be used when evaluating pain treatment in the early acute phase until 30 days after LSS. We have performed a Delphi procedure consisting of four rounds and an external endorsement round. A Dutch panel of 35 experts representing 10 stakeholder groups, including patient representatives, agreed to include five outcome domains in the COS. An international panel of 27 experts and the Dutch patient association for chronic pain endorsed the resulting COS. The COS developed consists of the following outcome domains: pain intensity, analgesic use, early mobilisation, length of stay, and adverse events. Six domains were excluded from the COS: patient satisfaction, quality of life, quality of recovery, daily functioning, physical function, and quality of sleep.

During the Delphi procedure, many arguments were raised in favour of or against the inclusion of various outcome domains, which are elaborated on in Data S2 and Data S3. The most notable result of these deliberations in the final COS is that the outcome domain patient satisfaction was excluded; with that, the COS contains no patient‐experience measures. This is in line with previously published COSs on low back pain and perioperative pain management (Boney et al. 2022; Chiarotto et al. 2015; Clement et al. 2015; Moonesinghe et al. 2019). The lack of validated and unambiguous measurement instruments of this outcome domain contributed to the exclusion of this domain. We acknowledge here that we were not following COMET guidance by starting with the domains first, followed by identifying suitable measurement instruments for the outcome domains. This is a limitation of the study and the development of this COS. It also highlights the need for instruments with sufficient measurement properties in this domain for future versions of this COS.

Similarly, the domain quality of life was excluded because the panel agreed it is unfeasible to measure in the early postoperative phase. This is consistent with previous literature, which states that although surgery should lead to improvement of quality of life in the long‐term, measurement of this domain in the early postoperative phase does not reflect the quality of life in the long‐term (Myles 2001). The increasingly highlighted domain quality of recovery did not reach consensus for inclusion in the COS. Although this domain was agreed to have great potential for measuring the multidimensionality of pain treatment and for early reflection of quality of life, the lack of experience on the clinical value of this domain led to exclusion from the COS (Borrell‐Vega, Humeidan, and Bergese 2018; Myles 2001). This is in contrast with a recent COS on peripheral regional anaesthesia techniques that did include the outcome domain quality of recovery (Hill et al. 2022) and an overarching COS for acute pain in general (Bova et al. 2023).

The current COS developed is a clinically relevant spine‐specific COS for the acute postoperative phase (until 30 days) and is a clear addition to the long‐term COS (Chiarotto et al. 2015; Clement et al. 2015). The relationship between short‐term and long‐term outcome measures should be explored in the future to help detect long‐term undesirable outcomes early, to improve continuous outcome monitoring, and to facilitate evaluation of quality of care. The domains included in the COS seem realistic for clinical use and reflect different dimensions of acute pain. This contributes to acceptability for uptake of this COS in future clinical and research practice. When implemented, an (inter)national registry can be populated. The outcomes of these registry data can be used to improve the COS and make it possible to conduct evaluations on the quality of care in order to ultimately improve acute pain management after LSS.

### 4.1 Strengths and Limitations

Limitations of this study need to be addressed. First, we opted to create a COS including outcome domains and corresponding measurement instruments. However, this is not in line with the COMET recommendations, stating the outcome domains should first be identified and only thereafter the identification of corresponding measurement instruments. If there are no (suitable) measurement instruments, these should be developed. In the current COS, this has resulted in an outcome domain being excluded due to the lack of validated and unambiguous measurement instruments, that is, the domain of patient satisfaction. Second, this study was designed as a national modified Delphi procedure, which could have introduced cross‐cultural bias. Therefore, an external endorsement round among international key opinion leaders was performed to explore potential cross‐cultural challenges for the implementation of the COS. This extra round did not reveal major issues, but cross‐cultural differences cannot be ruled out and need to be further elaborated, as the acceptance rate of 77% only barely achieved the cut‐off. Third, the largest group of respondents were orthopaedic spine surgeons, leading to one group being over‐represented in the panel composition. A multidisciplinary panel was intended, but participant sampling relied on responses of the stakeholders. This included orthopaedic spine surgeons and anaesthesiologists from Dutch spine‐related hospitals. Orthopaedic spine surgeons, representing the biggest group, were likely because this group has direct involvement and ultimate responsibility for spine surgeries, fostering a stronger sense of ownership in improving the quality of care for this patient group. Anaesthesiologists, by contrast, have broader roles, and no formal subspecialization in spine surgery exists. Although fewer anaesthesiologists participated, those who did had relevant expertise in this patient population. Recruiting nurses was even more challenging, as there is no specific registry for spine‐focused nurses in the Netherlands. As a result, we relied on snowball sampling and the project team's network to recruit five nursing stakeholders. Fourth, not all panellists could attend the live round, round 3. A fourth confirmation round was added to the procedure to create the opportunity for all panellists to provide feedback. This subsequently led to a supported COS among the entire panel with enhanced internal consistency. Fifth, the patient stakeholders included in the panel were familiar with medical language. This could have introduced bias in the form of exclusion of the perspective of lower‐educated patient representatives.

A main strength of this study is the performance of the Delphi process because of a number of reasons. First, the Delphi process was conducted within a short time frame to ensure that the knowledge and opinions remained fresh in the mind. Second, the live meeting was moderated strictly to persist on relevant discussions and to guarantee input from all panellists. Third, the panel included a wide variety of disciplines, capturing a multidisciplinary view. Fourth, we also included the patient perspective, with 8.5% being people with lived experience. This is on the lower end of what the COMET inventory on patient participation in Delphi procedures recommends (4%–50%) (Williamson et al. 2017).

The current COS only defined a COS of outcome domains. Future research should focus on establishing suitable corresponding measurement instruments, including the timing, context, and frequency of measurement, for example, by performing appropriate systematic literature reviews. These measurement instruments should be assessed for their content validity and additional psychometric properties according to the COSMIN recommendations (Prinsen et al. 2016).

## 5 Conclusions

To our knowledge, this is the first COS developed for evaluation of acute pain management after lumbar spine surgery. This minimal set of outcomes contains five outcomes This COS focuses on the first 30 postoperative days and is an addition to existing COSs for low back pain for long‐term outcomes. Future research should focus on establishing suitable corresponding measurement instruments. Furthermore, the implementation process should be conducted, and the COS should be evaluated and updated according to new research findings in order to establish continuous improvement of the COS.

## Author Contributions

All authors discussed the results and commented on the manuscript. I.H.W. conceptualisation, methodology, investigation, resources, data curation, writing – original draft, writing – review and editing, visualisation, project administration. K.C.P.V. conceptualisation, methodology, investigation, writing – review and editing, visualisation, supervision. M.G.E.F. conceptualisation, methodology, writing – review and editing. A.R. conceptualisation, methodology, investigation, writing – review and editing. R.L.M.B. writing – review and editing, visualisation, supervision. M.L.H. conceptualization, methodology, investigation, writing – original draft, writing – review and editing, visualisation, supervision.

## Conflicts of Interest

I.H.W., R.L.M.B., A.R., K.C.P.V., and M.G.E.F. declares no conflicts of interest. M.L.H. declares to receive grants or contracts from ZonMw ZE&GG, AO Foundation, and the Scoliosis Research Society. These grants are payments to the institution.

## Supporting information


Data S1.

Data S2.

Data S3.

## Data Availability

The authors confirm that the data supporting the findings of this study are available within the article and its Supporting Information. Additional data that support the findings of this study are available on request from the corresponding author, I.H.W.
