# Supplementary material for: Development of a Core Outcome Set of Domains to Evaluate Acute Pain Treatment After Lumbar Spine Surgery: A Modified Delphi Study
Source: Eur J Pain. 2025 Jan 13;29(2):e4784. doi: 10.1002/ejp.4784 (PMC11729254; doi:10.1002/ejp.4784)
Supplement: Supplementary file 1 — Data S1. [file EJP-29-0-s001.docx]

**Appendix 1: Characteristics of panel during consecutive Delphi rounds**

**Flow chart Delphi panel**

**
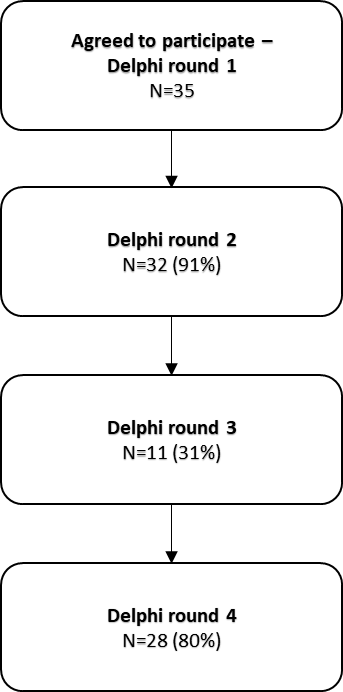
**

Figure 1: Flow chart of panel during the subsequent Delphi rounds.

**Delphi round 1 panel characteristics**

35 panelists completed round 1

The panelists were related to 14 different hospitals

Male/female ratio: 60/40%


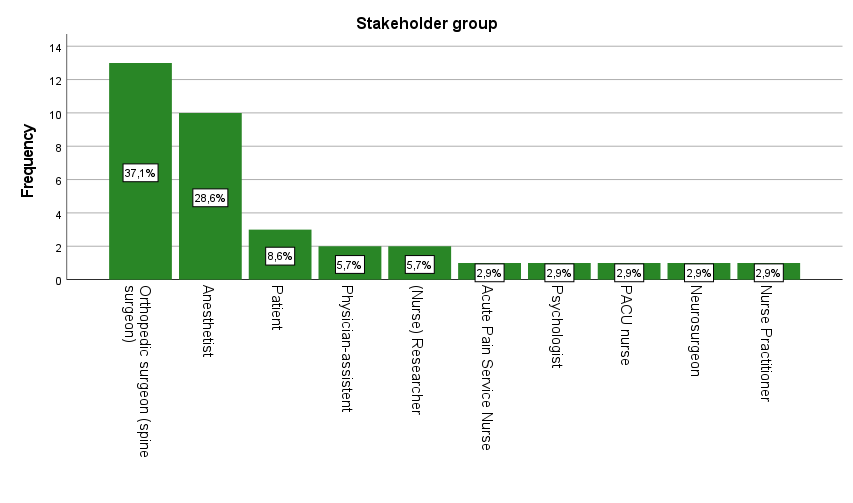


Figure 2: Frequency (%) of stakeholder groups represented in Delphi round 1 panel.


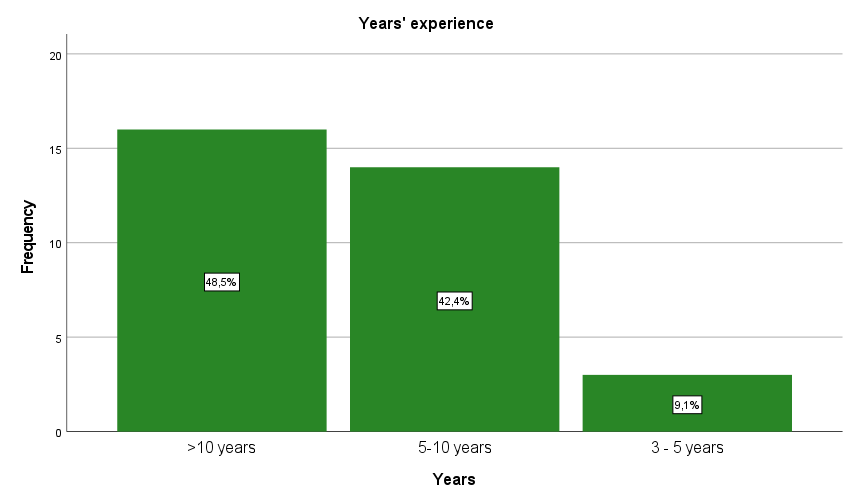


Figure 3: Frequency (%) of years of experience in Delphi round 1 panel.

**Delphi round 2 panel characteristics**

32/35 panelists completed round 2 (91%)

The panelists were related to 14 different hospitals

Male/female ratio: 56/44%


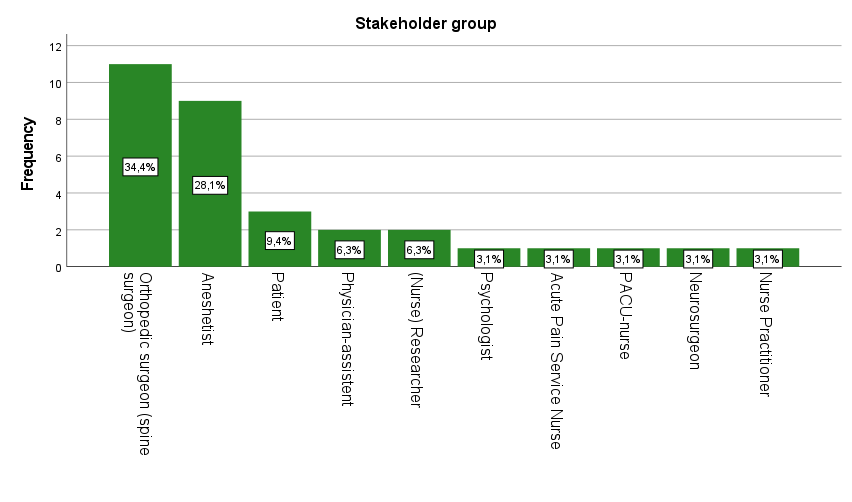


Figure 4: Frequency (%) of stakeholder groups represented in Delphi round 2 panel.


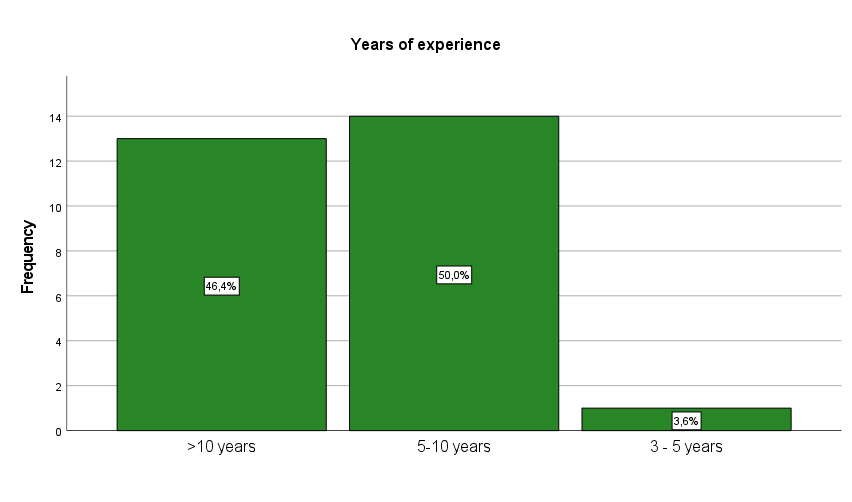


Figure 5: Frequency (%) of years of experience in Delphi round 2 panel.

**Delphi round 3 panel characteristics**

11/35 panelists attended the live meeting (31%)

The panelists were related to 8 different hospitals

Male/female ratio: 36/64%


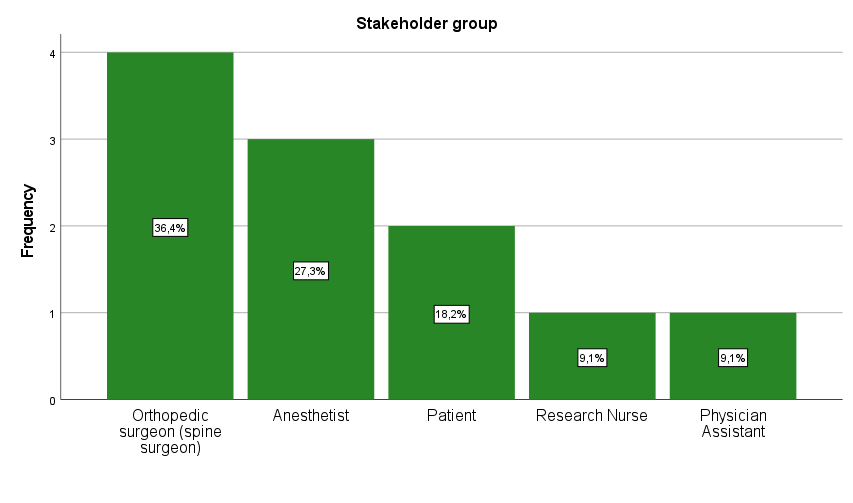


Figure 6: Frequency (%) of stakeholder groups represented in Delphi round 3 panel.

**Delphi round 4 panel characteristics**

28/35 panelists completed round 4 (80%)

The panelists were related to 8 different hospitals

Male/female ratio: 36/64%


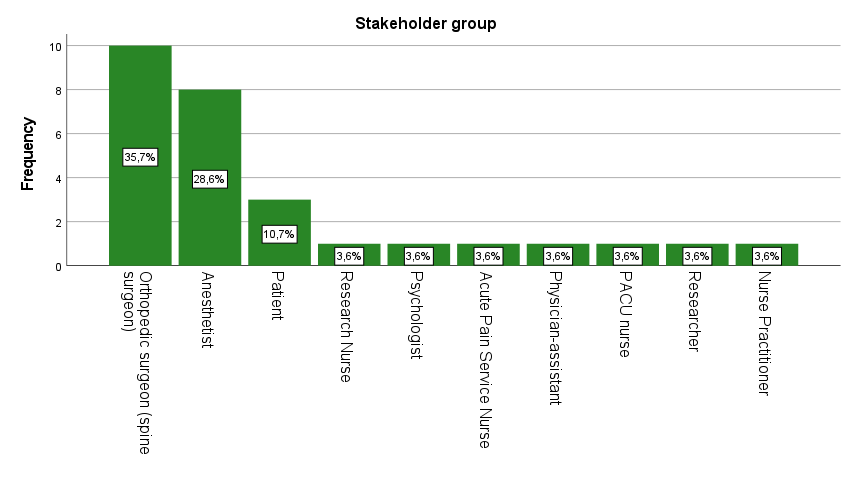


Figure 7: Frequency (%) of stakeholder groups represented in Delphi round 4 panel.


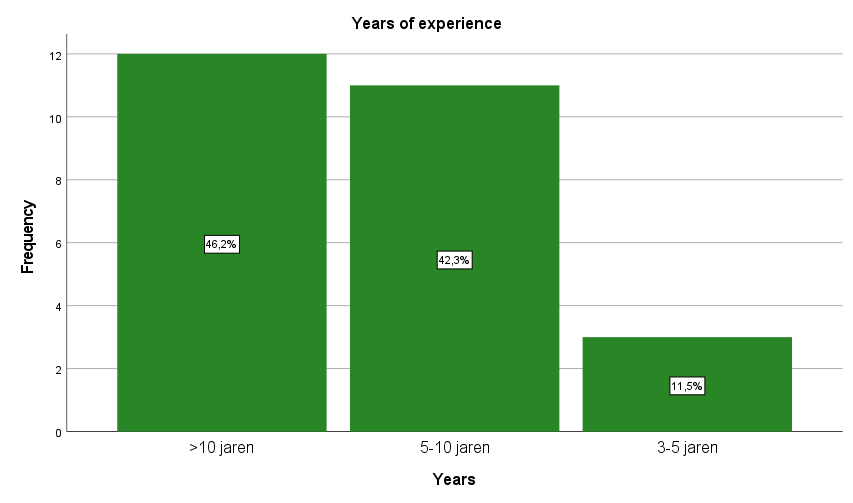


Figure 8: Frequency (%) of years of experience in Delphi round 4 panel.
