# Supplementary material for: Development of a Core Outcome Set of Domains to Evaluate Acute Pain Treatment After Lumbar Spine Surgery: A Modified Delphi Study
Source: Eur J Pain. 2025 Jan 13;29(2):e4784. doi: 10.1002/ejp.4784 (PMC11729254; doi:10.1002/ejp.4784)
Supplement: Supplementary file 2 — Data S2. [file EJP-29-0-s002.docx]

**Appendix 2: Selection process of outcome domains per Delphi round**

**Delphi round 1**


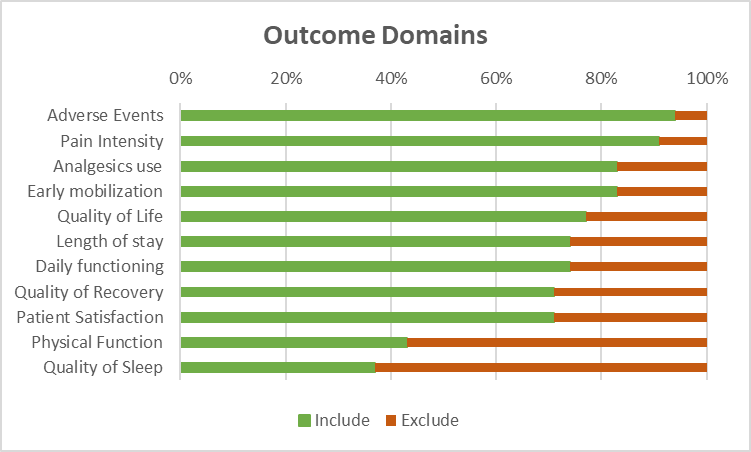


Open remarks:

- *“Early mobilization is dependent on the type of patient and preoperative functioning.”*
- Quality of life:
  - *“Most of the questions being asked here may be a bit premature, especially so shortly postoperatively.”*
  - *"I believe that achieving QoL in the short term is possible but not relevant. This is truly too short a time span to assess this. Many domains in the questionnaire patients have not yet reached. In my opinion, this pertains to the longer term.”*
  - *"It seems to me that the first 30 days are still too early."*
- Patient satisfaction:
  - *"Please note, after spine surgery, it can definitely take longer than 30 days before the patient is satisfied and considers whether they would do it again. It seems too early to ask these kinds of questions. When it comes to pain management, I understand, but undergoing treatment should be taken with a grain of salt <30 days postoperatively."*
  - *"It is greatly influenced by the 'experience' people have: how much they like others, their own personality, sympathy towards doctors/healthcare providers, etc."*
  - *"I believe measuring satisfaction is still too early in the early postoperative phase."*
- Physical function: *“Certainly, for fusion surgery, the first 30 days are still too early for this, as it does not provide meaningful information.”*

**Delphi round 2**


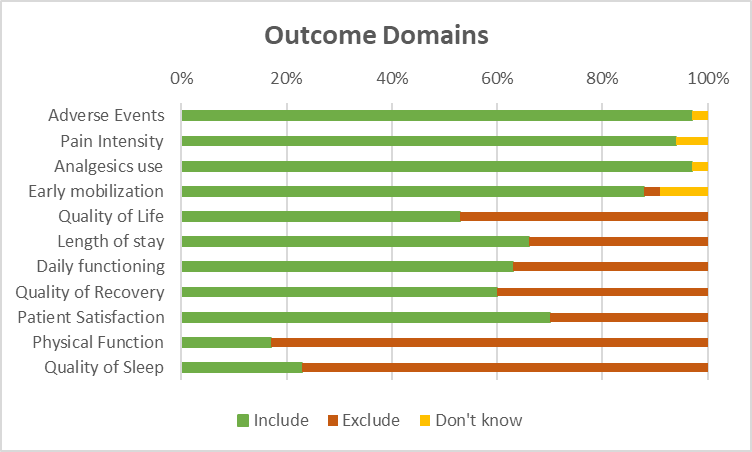


Open remarks:

- Adverse events: *"I find it difficult in this domain to consistently distinguish whether it reflects something about pain management or about the surgery itself."*
- Analgesics use: *“Patients also use painkillers for other issues, making it a challenging tool to assess interventions.”*
- Early mobilization:
  - *"I believe that mobilization is a very good indicator of early postoperative function."*
  - *"In my opinion, it is determined by so many factors, including preoperative condition and comorbidities, that it may not provide much insight into the quality of the postoperative course. So, mobilization itself may be important, but whether it is early or not may not be so relevant in my view. It could influence the level of pain, but this is also dependent on many factors. In my experience, people with a lot of pain tend to mobilize less later, while those with less pain mobilize more and sooner. It is more like this than the other way around, so is it important to measure it?"*
  - *"This aspect is important, but it is influenced by many factors, so it may not always be straightforward?"*
- Daily functioning:
  - *“These outcome measures are not applicable in the early postoperative period. But it is also strongly dependent on the type of surgery. When looking at (semi-acute) hernia repair, it is important to look at return-to-work. However, with an extensive decompression, 30 day daily recovery is less relevant. It is however good to look at ADL independence, even though this may only be to look at time/costs consuming of care.”*
  - *“Many activities described in this domain are strongly discouraged with spine surgery in the first 6 weeks postoperatively.”*
  - *“In the early postoperative phase this domain does not seem feasible, however, for the long-term it is.” (n=3)*
- Quality of life:
  - *“In the early postoperative phase patients often have a lot of pain; mobilization is still limited and revalidation ongoing. Determining quality of life is pointless in the early postoperative phase. Above all, the goal of spine surgery is not to improve quality of life in the first postoperative days, but for the years afterwards.”*
  - *“It is too early to ask for quality of life.” (n=5)*
  - *“It is extremely important, however, maybe too soon. On the other hand, if we measure quality of life early on, maybe a trend can be visualized for a population who recovers quickly, which can have consequences for the follow-up treatment plan.”*
- Quality of recovery: *“I think the quality of recovery provides a broad view on recovery. However, the association with pain is limited as other factors also play a role on the recovery. Therefore I do not think we should use this domain to evaluate pain treatment.”*
- Length of stay:
  - *“Length of stay is dependent on many factors.”*
  - *“When talking about cost-effectiveness of a pain treatment this can be a relevant indicator.”*
- Patient satisfaction:
  - *"Please note, after spine surgery, it can definitely take longer than 30 days before the patient is satisfied and considers whether they would do it again. It seems too early to ask these kinds of questions. When it comes to pain management, I understand, but undergoing treatment should be taken with a grain of salt <30 days postoperatively."*
  - *“I don’t think it is useful to say something about patient satisfaction that early on after surgeries.”*
  - *“I think you should specify very clearly what type of satisfaction you want to measure: about pain treatment, surgery, service, etc.”*
  - *“Satisfaction is influenced by many factors which have nothing to do with the pain treatment of a specific patient.”*
- Physical function: *“Too early to measure.”*
- Quality of sleep:
  - *“Sleep is extremely important for postoperative recovery, but I think that a hospital admission has a disruptive effect on the quality of sleep that it will not matter in comparison of different pain interventions. It is dependent on many factors.”*
  - *“In a hospital sleep is disrupted by many factors.” (n=2)*
  - *“Sleep is disrupted by anesthesia in the first few days. In the first days there may not be a deep sleep mode.”*
  - *“People who claim to have high pain intensity may have a good quality of sleep.”*
  - *“Sleep determines the quality of recovery. When paying attention to this, this seems relevant in the early postoperative phase.”*
  - *“I would link this domain to another domain in terms of measuring pain interference with quality of sleep.”*

**Delphi round 3**


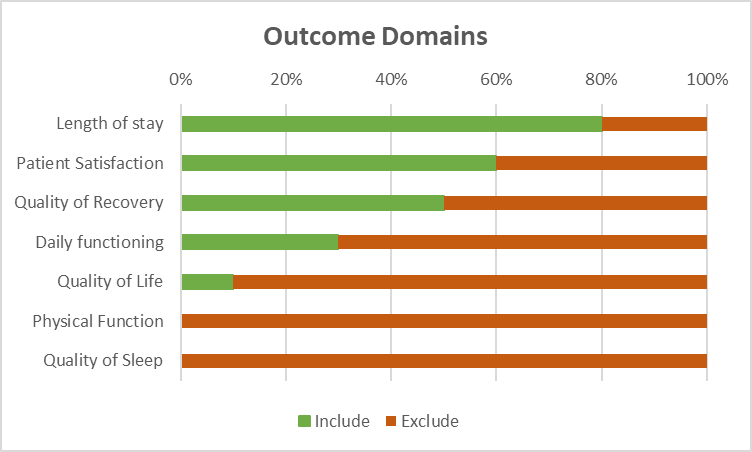


Summary of discussion on the outcome domains:

- See Appendix 3, table A3.1 for a summary of the discussions of the included domains.
- Patient satisfaction: does this domain say something about satisfaction with the pain treatment, or other factors such as service, surgery outcome? Furthermore, the first 30 days is too early to measure this domain. If someone has a high pain intensity score, then had an intervention, and then measurement of satisfaction, this can still be scored low. It is not exactly valid to measure pain treatment effectiveness. However, it is relevant to know how satisfied a patient is. The measurement instruments available to measure this domain are not valid to measure what we want to know.
- Quality of recovery: this domain is too young and unknown, but an interesting domain to explore for future versions of this core outcome set. A correlation between early quality of recovery and long-term quality of life should be explored. The goal of this Delphi procedure was to make a clinically applicable COS, but this domain is not yet investigated in the setting of lumbar spine surgery. There is too little experience with this domain to be included in the COS.
- Daily functioning: it is too early to measure daily functioning within 30 days after surgery. It says something about the quality of recovery, but little about specific pain interference. It is influenced by too many factors next to pain. Does not contribute much.
- Quality of life: although this domain is thought to be important, and influenced by pain, the early postoperative phase is considered too early to measure this domain.
