# Supplementary material for: Development of a Core Outcome Set of Domains to Evaluate Acute Pain Treatment After Lumbar Spine Surgery: A Modified Delphi Study
Source: Eur J Pain. 2025 Jan 13;29(2):e4784. doi: 10.1002/ejp.4784 (PMC11729254; doi:10.1002/ejp.4784)
Supplement: Supplementary file 3 — Data S3. [file EJP-29-0-s003.docx]

**Appendix 3. Discussion topics on included items in the Core Outcome Set**

Table A3.1 **Discussion topics on outcome domains and measurement instruments from the final core outcome set**

| **Domains** | **Discussion on outcome domain from Delphi procedure supplemented by literature** | **Discussion on proposed measurement instruments from Delphi procedure supplemented by literature (if applicable)** |
| --- | --- | --- |
| Pain intensity | This domain was deemed important for evaluation of acute pain management. Treating pain as the fifth vital sign has been abandoned now as it may result in increased opioid analgesic use and length of stay whilst not improving outcome[1,5]. However, pain scores are widely available and used, and can contribute to an overall understanding of the patient’s status when combined with other outcome domains[2,7,10,12]. | The *Numeric Rating Scale (NRS)* as corresponding measurement instrument is a simple and responsive scale which can be used in all settings and therefore improves clinical implementation of this COS[8]. It is a globally applied and widely available, easy measurement of first pain experience after surgery.  Although the relevance of a more extensive pain assessment tool was highlighted to assess multiple aspects of pain intensity, e.g. the Brief Pain Inventory, these tools were assumed to be too extensive and unfeasible for patients short after surgery. The panel agreed that the other included outcome domains in the COS sufficiently cover the multidimensionality. |
| Analgesic use | Analgesic use should be reported for different types of analgesics (e.g. opioid vs non-opioid). Experts agreed to also carefully take into account preoperative opioid use, because this impacts the postoperative need for analgesics[14]. | The measure for *analgesic use yes or no* can be used a starting question for measuring analgesic use, e.g. at 30 day follow-up. If analgesics are used at this time point, other measures for analgesic use can be taken.  As such, the *frequency of analgesic use* can be reported. This is the number of doses within a certain time period, e.g. in 24 hours. *The total amount of analgesics during this time period* represents the cumulative dose of one or more analgesics. It can be convenient to report dose and frequency for each type of analgesic, especially opioid versus non-opioid. Initiatives for a Medication Quantification Scale have been taken in which the amount and type of pain medications prescribed can be quantified into a total score[6]. This scale has not been widely accepted but the concept could be interesting to explore. |
| Early mobilization | Poor pain control impairs early mobilization[11]. Impaired mobilization can be an indicator to adjust pain. Enhanced Recovery After Surgery (ERAS) protocols strongly advise encouragement of early mobilization in LSS despite low grade evidence[3]. Early mobilization is linked to reduced morbidity and length of stay[4]. | The instrument *clinically relevant mobilization* is defined more clearly and uniformly among different hospitals and countries by using Enhanced Recovery After Surgery (ERAS) protocols, as these have defined the degree of mobilization a patient has to achieve before hospital discharge. Shields et al. [13] presented an ambulation protocol after lumbar fusion surgery, in which they advise the following:   1. sitting on the edge of the bed on the night of surgery; 2. physical therapy on two occasions on the first postoperative day; 3. walking in the hall and climbing stairs on the second postoperative day.   However, these measures have not been fully researched in terms of applicability and measurement properties and are therefore input for future research.  Measuring instrument *time to return to daily activities (ADL)* is included in domain *early mobilization* by the project team. It is a reflection of out-of-hospital mobilization until 30 days, and a possible link to daily functioning on the long-term. Research is necessary to analyze the relation between this short- and long-term parameters. |
| Length of stay | Lengthened hospital stay is associated with all other included outcome domains in the COS – and is therefore multifactorial [4]. It is also an indicator for quality of care, a process indicator for evaluation of cost-effectiveness and healthcare consumption. These are relevant parameters because of the societal burden of healthcare and shortage in healthcare personnel. | Discussion was held on if length of stay should be reported in days or hours. Reporting in hours is more prone to delays in logistics rather than poor pain control. However, to increase hospital stay with an extra night can actually be an indicator for insufficient pain control. Therefore, the panel choose to report length of stay in days. |
| Adverse events | Adverse events should be reported until at least 30 days after surgery[9]. | A global classification system for adverse events and side-effects which is applicable for anesthetic as well as surgical purposes should be developed. |

**References**

[1] Baamer RM, Iqbal A, Lobo DN, Knaggs RD, Levy NA, Toh LS. Utility of unidimensional and functional pain assessment tools in adult postoperative patients: a systematic review. Br J Anaesth 2022;128:874–888.

[2] Clement RC, Welander A, Stowell C, Cha TD, Chen JL, Davies M, Fairbank JC, Foley KT, Gehrchen M, Hagg O, Jacobs WC, Kahler R, Khan SN, Lieberman IH, Morisson B, Ohnmeiss DD, Peul WC, Shonnard NH, Smuck MW, Solberg TK, Stromqvist BH, Hooff MLV, Wasan AD, Willems PC, Yeo W, Fritzell P. A proposed set of metrics for standardized outcome reporting in the management of low back pain. Acta Orthop 2015;86:523–533.

[3] Debono B, Wainwright TW, Wang MY, Sigmundsson FG, Yang MMH, Smid-Nanninga H, Bonnal A, Le Huec JC, Fawcett WJ, Ljungqvist O, Lonjon G, de Boer HD. Consensus statement for perioperative care in lumbar spinal fusion: Enhanced Recovery After Surgery (ERAS®) Society recommendations. Spine Journal 2021;000.

[4] Epstein NE. A review article on the benefits of early mobilization following spinal surgery and other medical/surgical procedures. Surg Neurol Int 2014;5.

[5] Frasco PE, Sprung J, Trentman TL. The impact of the joint commission for accreditation of healthcare organizations pain initiative on perioperative opiate consumption and recovery room length of stay. Anesth Analg 2005;100:162–168.

[6] Gallizzi MA, Khazai RS, Gagnon CM, Bruehl S, Harden RN. Use of a Medication Quantification Scale forComparison of Pain Medication Usage inPatients with Complex Regional PainSyndrome (CRPS). Pain Medicine (United States) 2015;16:494–500.

[7] Hill J, Ashken T, West S, MacFarlane AJR, El-Boghdadly K, Albrecht E, Chin KJ, Fox B, Gupta A, Haskins S, Haslam N, Hogg RMG, Hormis A, Johnston DF, Mariano ER, Merjavy P, Moll T, Parry J, Pawa A, Russon K, Sebastian MP, Turbitt L, Womack J, Chazapis M. Core outcome set for peripheral regional anesthesia research: a systematic review and Delphi study. Reg Anesth Pain Med 2022;47:691–697.

[8] Hjermstad MJ, Fayers PM, Haugen DF, Caraceni A, Hanks GW, Loge JH, Fainsinger R, Aass N, Kaasa S. Studies comparing numerical rating scales, verbal rating scales, and visual analogue scales for assessment of pain intensity in adults: A systematic literature review. J Pain Symptom Manage 2011;41:1073–1093.

[9] Mizushima T, Yamamoto H, Marubashi S, Kamiya K, Wakabayashi G, Miyata H, Seto Y, Doki Y, Mori M. Validity and significance of 30-day mortality rate as a quality indicator for gastrointestinal cancer surgeries. Ann Gastroenterol Surg 2018;2:231–240.

[10] Pogatzki-Zahn EM, Liedgens H, Hummelshoj L, Meissner W, Weinmann C, Treede RD, Vincent K, Zahn P, Kaiser U. Developing consensus on core outcome domains for assessing effectiveness in perioperative pain management: results of the PROMPT/IMI-PainCare Delphi Meeting. Pain 2021;162:2717–2736.

[11] Rivas E, Cohen B, Pu X, Xiang L, Saasouh W, Mao G, Minko P, Mosteller L, Volio A, Maheshwari K, Sessler DI, Turan A. Pain and Opioid Consumption and Mobilization after Surgery: Post Hoc Analysis of Two Randomized Trials. Anesthesiology 2022;136:115–126.

[12] Rothaug J, Zaslansky R, Schwenkglenks M, Komann M, Allvin R, Backström R, Brill S, Buchholz I, Engel C, Fletcher D, Fodor L, Funk P, Gerbershagen HJ, Gordon DB, Konrad C, Kopf A, Leykin Y, Pogatzki-Zahn E, Puig M, Rawal N, Taylor RS, Ullrich K, Volk T, Yahiaoui-Doktor M, Meissner W. Patients’ perception of postoperative pain management: Validation of the international pain outcomes (IPO) questionnaire. Journal of Pain 2013;14:1361–1370.

[13] Shields L, Clark L, Glassman S, Shields C. Decreasing hospital length of stay following lumbar fusion utilizing multidisciplinary committee meetings involving surgeons and other caretakers. Surg Neurol Int 2017;8.

[14] Yerneni K, Nichols N, Abecassis ZA, Karras CL, Tan LA. Preoperative Opioid Use and Clinical Outcomes in Spine Surgery: A Systematic Review. Neurosurgery 2020;86:E490–E507.
